# Abernethy Malformation Type Ib in a Patient With Trisomy 21: A Rare Case of Portal Vein Absence, Mesenteric Thrombosis, and Bowel Perforation

**DOI:** 10.7759/cureus.99481

**Published:** 2025-12-17

**Authors:** Christian Benignus, Julian Müller-Kühnle, Miruna Popescu, Benjamin Goeppert, Thomas Schiedeck

**Affiliations:** 1 Department of General Surgery, Ludwigsburg Hospital, Regionale Kliniken Holding und Services GmbH (RKH), Ludwigsburg, DEU; 2 Department of General Surgery, Paracelsus Medical University, Salzburg, AUT; 3 Department of General Internal Medicine and Nephrology, Robert Bosch Krankenhaus, Stuttgart, DEU; 4 Institute of Pathology, Ludwigsburg Hospital, Regionale Kliniken Holding und Services GmbH (RKH), Ludwigsburg, DEU

**Keywords:** abernethy malformation, congenital portosystemic shunt, down snydrome, open abdomen treatment, portal vein absence, trisomy 21

## Abstract

Congenital portosystemic shunts (CPSSs), also known as Abernethy malformations, are rare vascular anomalies in which blood from the portal circulation bypasses the liver and drains directly into the systemic circulation. They are classified into type I, involving complete diversion of portal blood with absent intrahepatic portal branches, and type II, where partial shunting occurs with preserved intrahepatic perfusion. While often asymptomatic, these malformations can cause diverse complications, including hepatic encephalopathy, pulmonary hypertension, and gastrointestinal bleeding. We present the case of a 21-year-old woman with trisomy 21 who developed a severe and multifaceted clinical course due to an undiagnosed Abernethy malformation type Ib. She was initially referred for transjugular intrahepatic portosystemic shunt (TIPS) placement after imaging revealed thromboses of the mesenteric and splenic veins, splenomegaly, and signs of portal hypertension. Endoscopic findings confirmed grade II esophageal varices and portal hypertensive gastropathy. Imaging showed thrombosis of the splenic vein (SV) and superior mesenteric vein (SMV), with drainage into the inferior vena cava (IVC) and absence of a native portal vein, consistent with Abernethy malformation type I. The planned intervention was not feasible due to anatomical constraints and extensive thrombosis. The patient was started on full anticoagulation. Three days later, she developed acute abdominal symptoms. Imaging revealed dilated, thickened bowel loops with signs of obstruction. An exploratory laparotomy uncovered a conglomerate mass with intestinal perforation and purulent peritonitis. Resection and double anastomosis were performed, but due to massive edema, initial closure was impossible, and open abdomen treatment was required. She developed systemic inflammatory response syndrome, acute kidney injury, and bilateral pleural effusions. Targeted antimicrobial therapy was initiated based on microbiological cultures. After 26 days in intensive care, she stabilized and was transferred to the general ward. Histological evaluation of the resected specimen revealed transmural colonic damage, intestinal thromboses, and IgG4-positive lymphadenopathy without evidence of malignancy or systemic IgG4 disease. Follow-up imaging showed persistent thromboses without progression. Compared to published cases, this is the only report describing the combination of trisomy 21, Abernethy malformation type Ib, mesenteric thrombosis, bowel perforation, sepsis, and IgG4-positive lymphadenopathy in one patient. No interventional treatment options were available, and management was limited to surgical and supportive care. This case illustrates a rare and severe manifestation of CPSS with complex vascular and immunologic features. The risk of future complications remains high, and liver transplantation may represent the only curative approach in selected patients with Abernethy malformation type I.

## Introduction

Trisomy 21 (also known as Down syndrome) is the most common chromosomal abnormality among liveborn infants and is associated with distinct dysmorphic features, varying degrees of intellectual disability, and a wide range of congenital malformations [1, 2]. In addition to the well-known cardiac defects (such as atrioventricular septal defects) and gastrointestinal anomalies (like duodenal atresia and Hirschsprung disease), individuals with trisomy 21 may also present with rare vascular abnormalities [3]. Among these, congenital portosystemic shunts (CPSS) represent a particularly unusual but clinically significant finding [4]. The overall incidence of CPSS is estimated at approximately 1 in 30,000 live births, with persistent shunts occurring in about one in 50,000 individuals [5].

CPSS, also referred to as Abernethy malformations, are characterized by an abnormal vascular connection between the portal venous system and the systemic circulation, allowing blood from the intestines to bypass the liver. This abnormal shunting can interfere with hepatic metabolism and detoxification processes [5]. The malformations are classified into two primary types based on the extent of portal blood diversion [6].

Type I involves a complete diversion of portal blood away from the liver due to the absence of intrahepatic portal venous branches. This type is further subdivided into type Ia, in which the superior mesenteric vein (SMV) and splenic vein (SV) drain separately into the systemic venous system, and type Ib, where these veins first join to form a short extrahepatic portal vein before connecting to systemic circulation.

Type II is defined by a partial shunt in which intrahepatic portal branches are present. In this configuration, a portion of portal blood reaches the liver, while another portion is diverted through a side-to-side anastomosis with the systemic circulation. The clinical presentation can vary depending on the shunt size and hepatic involvement.

Abernethy malformations can have significant multisystem consequences, including hepatic encephalopathy, hyperammonemia, growth retardation, pulmonary hypertension, and even cardiac or neurological complications. Early diagnosis and individualized treatment strategies are essential, particularly in syndromic patients who may already have multiple comorbidities [7, 8].

Here, we describe the case of an adult patient with confirmed trisomy 21 who developed a severe clinical course due to a previously undiagnosed Abernethy malformation. The condition led to prolonged intensive care management and a multidisciplinary therapeutic approach. This case emphasizes the importance of considering rare vascular anomalies such as CPSS in the differential diagnosis when patients with Down syndrome present with unexplained hepatic or neurocognitive symptoms.

## Case presentation

A 21-year-old female patient with trisomy 21 was referred to our hospital from an external clinic for placement of a transjugular intrahepatic portosystemic shunt (TIPS) due to suspected portal vein thrombosis, mesenteric vein thrombosis, and splenomegaly. The indication for TIPS was based on radiological findings and progressive signs of portal hypertension. Clinically, the patient presented with diffuse abdominal pain, persistent nausea, a visibly distended abdomen, and widespread tenderness to palpation. Her vital signs were stable on admission, with no signs of peritonitis. Laboratory studies showed mildly elevated inflammatory markers and mild anemia. An overview of baseline laboratory parameters is provided in Table 1.

**Table 1:** Initial blood parameters at first presentation CRP: C-reactive protein; INR: international normalized ratio

Parameter	Result	Reference Range
Leukocytes	12.2 × 10^3^/µl	4.3-10 × 10^3^/µl
Hemoglobin	10.3 g/dl	12.3-15.3 g/dl
Platelet count	196 × 10^3^/µl	150-400 × 10^3^/µl
INR	1.64	0.7-1.3
Sodium	132 mmol/l	135-145 mmol/l
Potassium	3.9 mmol/l	3.5-5.1 mmol/l
Total bilirubin	0.6 mg/dl	<1.3 mg/dl
CRP	36.4 mg/l	<5 mg/l
Albumin	34.8 g/l	35-52 g/l
Procalcitonin	0.08 ng/ml	<0.05 ng/ml
Interleukin-6	15.7 pg/ml	<7 pg/ml

**Figure 1:**
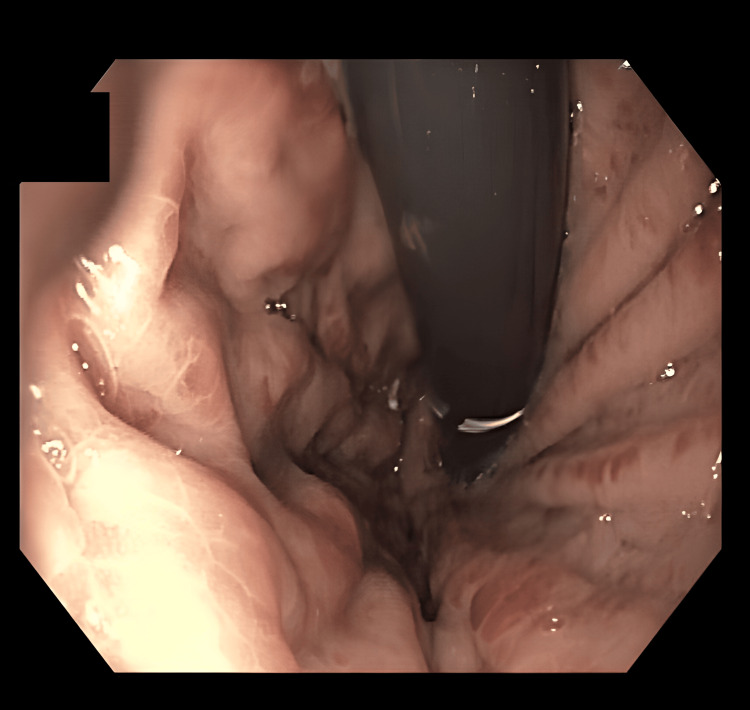
The esophagogastroduodenoscopy (EGD) shows signs of portal hypertensive gastropathy

**Figure 2:**
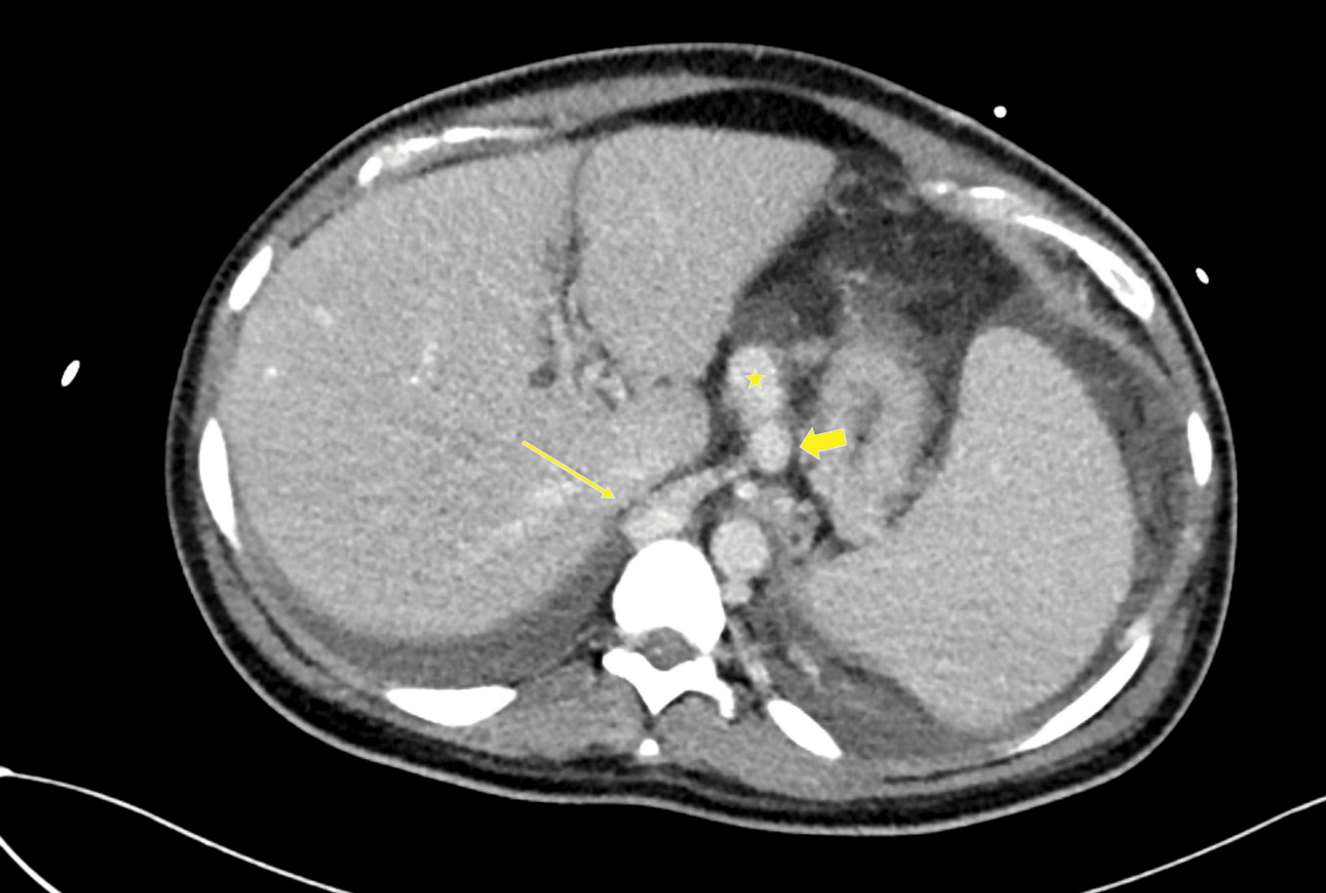
Axial CT scan showing the Abernethy malformation type Ib Thin arrow: inferior vena cava; thick arrow: splenic vein; asterisk: superior mesenteric vein

**Figure 3:**
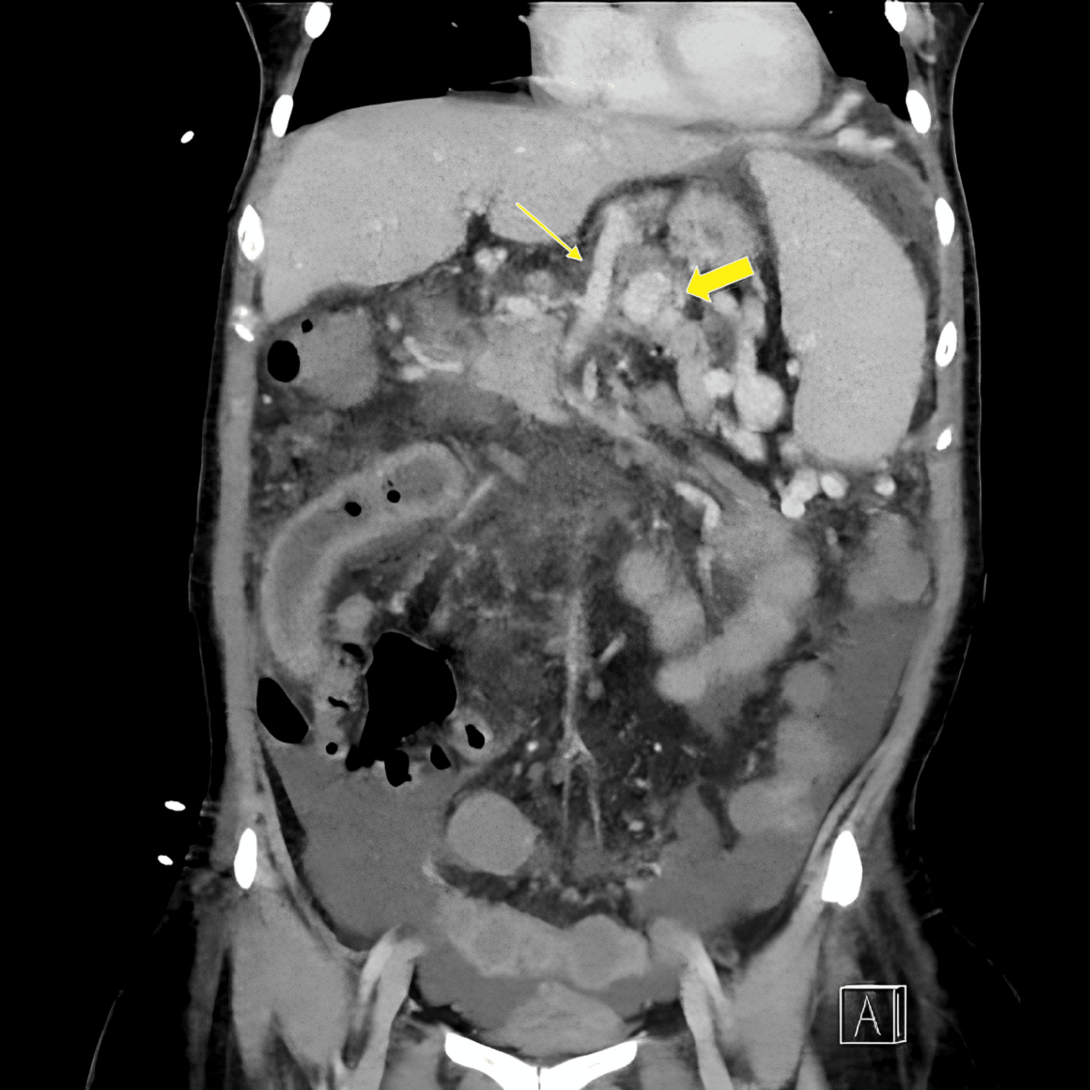
Frontal CT scan showing ascites in the lower abdomen and thickened small bowel loops in the right mid-abdomen. The superior mesenteric vein and splenic vein extend far cranially. The portal vein is absent (Thin arrow: Superior mesenteric vein, thick arrow: Splenic vein).

An esophagogastroduodenoscopy (EGD) revealed grade II esophageal varices and signs of portal hypertensive gastropathy, indicating relevant portal hypertension (Figure 1). Subsequent contrast-enhanced computed tomography (CT) demonstrated the absence of a recognizable portal vein, a long-segment, nearly occlusive thrombosis of the SMV with partially occluded distal branches, and complete thrombosis of the SV. Prominent venous collaterals indicated extensive portosystemic shunting. Both the SMV and the SV drained together directly into the inferior vena cava (IVC), consistent with an Abernethy malformation type Ib, an extremely rare congenital vascular anomaly. The small bowel loops appeared edematous and thickened, suggestive of venous congestion. Ascites was present in the lower abdomen, further supporting the clinical suspicion of advanced portal hypertension (Figures 2, 3).

Given the absence of a patent portal vein and the congenital vascular anomaly, the planned TIPS procedure and thrombolysis were deemed technically unfeasible. As an alternative, a weight-adjusted full anticoagulation with low molecular weight heparin (LMWH) was initiated to prevent further thrombotic progression. The patient was later transitioned to oral anticoagulation with apixaban. Additionally, carvedilol was recommended for portal pressure reduction; however, initiation was deferred due to episodes of symptomatic hypotension.

Three days after admission, the patient developed acute worsening of abdominal symptoms, with increasing pain and abdominal distension. A bedside abdominal ultrasound suggested small bowel ileus. Repeat CT imaging revealed a pronounced caliber change in the right lower quadrant, with small bowel loops distended up to 5 cm and several markedly thickened intestinal segments, concerning for a mechanical obstruction.

Urgent surgical exploration via midline laparotomy was performed. Intraoperatively, the small bowel loops were found to be massively thickened and dilated due to a firm, infiltrative conglomerate mass in the right lower abdomen. This mass encompassed several loops of ileum as well as the ascending colon. In the region of the ascending colon, a transmural perforation with purulent peritonitis was identified. The vermiform appendix and cecum appeared normal. Extensive adhesiolysis and resection of the involved intestinal segments were performed. A jejuno-ileostomy was created using an end-to-end technique, and an ileotransversostomy was fashioned using a side-to-side technique. Due to marked intra-abdominal edema and concern for compartment syndrome, the abdomen could not be closed primarily. The patient was managed using open abdomen treatment with temporary abdominal closure.

Four days later, she underwent planned surgical revision with definitive abdominal wall closure. Multiple pathogens were identified during the ICU stay and managed with targeted antimicrobial therapy.

Bilateral pleural effusions required chest drainage, and sepsis-related acute kidney injury (AKI) with fluctuating urine output was managed with intermittent diuretics.

After 26 days in the ICU, the patient was clinically stabilized and transferred to a general medical ward. With normalization of inflammatory parameters and a stable respiratory situation, the antimicrobial therapy was discontinued. Chest drains were removed, oral nutrition was reintroduced and well tolerated, and pain management was gradually tapered. The patient was discharged home in good condition with continued full-dose anticoagulation using apixaban.

**Figure 4:**
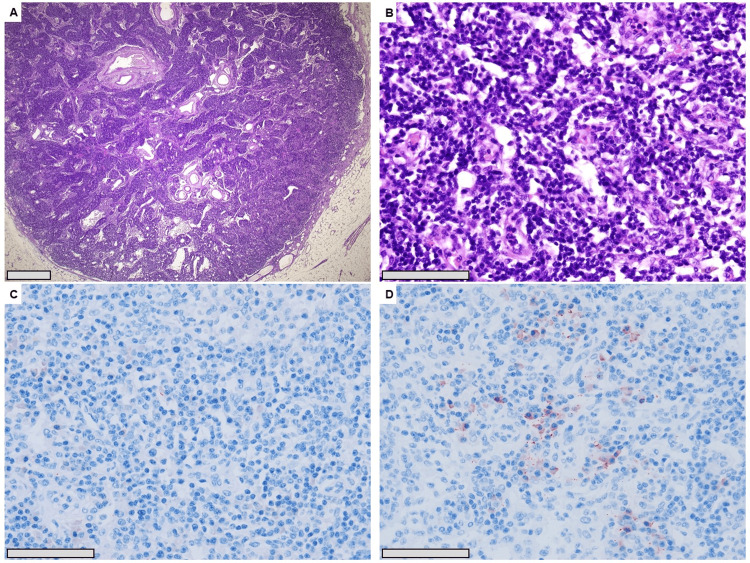
Histopathological and immunohistochemical findings A. Scanning magnification shows an enlarged lymph node with an expanded interfollicular zone, dilated sinuses, and sinus histiocytosis (HE, 20x); B. A higher power view highlights significantly increased plasma cells in the lymph node parenchyma (HE, 400x); C. IgG IHC shows scattered, weakly IgG-positive plasma cells (IHC, 400x); D. In comparison, there are numerous IgG4-positive plasma cells (50/HPF in this field) in the lymph node paracortex (IHC, 400x). Scale bar for A = 1000 µm; for B, C, and D = 100 µm. IHC: immunohistochemistry; HPF: high-power field

Histopathological analysis excluded malignancy. A transmural colonic defect with hemorrhage and thrombosis in segments of the small intestine was confirmed. Enlarged mesenteric lymph nodes showed sinus ectasia with dense plasma cell infiltration, including numerous IgG4-positive plasma cells, but without light-chain restriction. Immunohistochemistry supported reactive changes with histiocytic involvement. Overall, the findings were interpreted as IgG4-associated lymphadenopathy, although serum IgG4 levels remained normal (Figure 4).

At a three-month follow-up, the patient was in stable clinical condition with no signs of infection or gastrointestinal symptoms. Follow-up imaging, however, showed persistent thrombosis of the portal and mesenteric venous systems, without progression. Further multidisciplinary management and long-term follow-up were recommended due to the complex vascular and immunological background.

## Discussion

Our case describes a 21-year-old woman with trisomy 21 and an Abernethy malformation type Ib, complicated by extensive thrombosis of the mesenteric and splenic veins, significant portal hypertension, bowel ischemia with perforation, and polymicrobial sepsis. Compared to published reports, this case represents a uniquely complex and multifactorial presentation of a CPSS, demonstrating features not previously described in combination.

In the case reported by Yao et al., an 18-year-old female patient with a type IIC shunt developed gastrointestinal bleeding due to portal hypertension in the absence of intrahepatic portal vein branches. The clinical course was managed successfully with endoscopic therapy and beta-blockers. Although portal hypertension is not a typical finding in Abernethy malformation, their case and ours both highlight its occurrence in specific anatomical contexts. However, the patient described by Yao remained stable and did not develop thrombosis, ischemia, or systemic infection [9].

Agarwal et al. described a young adult with type II Abernethy malformation and severe pulmonary hypertension, which improved markedly following endovascular closure of the shunt. Their case emphasizes the reversibility of certain CPSS complications when interventional treatment is feasible. In contrast, our patient’s complete end-to-side shunt (type Ib) and absence of a native portal vein excluded closure strategies such as embolization or placement of a TIPS. Moreover, the clinical course in our patient was dominated not by cardiopulmonary symptoms but by gastrointestinal compromise and systemic inflammation [10].

Nohomovich et al. presented a patient with trisomy 21 and CPSS who developed hyperammonemia and choreiform movements, again successfully treated via endovascular closure. This case reinforces the syndromic association between trisomy 21 and CPSS and supports early consideration of shunt pathology in this population. However, their case followed a metabolic-neurologic trajectory, whereas ours was shaped by progressive splanchnic thrombosis, bowel necrosis, and a pronounced septic component [11].

The report by Kayedi et al. focused on a 27-year-old woman with Abernethy malformation type Ib that was initially misdiagnosed as chronic portal vein thrombosis, a diagnostic challenge that mirrors our own experience. Their case underscores the importance of careful vascular imaging in distinguishing congenital from acquired vascular pathology. However, their patient remained clinically stable, without the secondary complications seen in our case [12].

The most recent report by Li et al. described a portosystemic shunt between the inferior mesenteric vein and the right ovarian vein in a patient presenting with gastrointestinal bleeding. Their literature review identified 34 patients with Abernethy malformation and gastrointestinal bleeding, most of whom had type II shunts. This supports the growing recognition that gastrointestinal bleeding can be a prominent and sometimes initial manifestation of CPSS. The authors concluded that surgical or endovascular closure of the shunt is effective in most cases, although high-flow shunts may be resistant to coil embolization alone. In our case, however, interventional treatment was not an option due to both the anatomy (type Ib with absent portal vein) and the extensive thrombosis, again highlighting a key distinction in therapeutic feasibility [13].

Pathophysiologically, our case illustrates the severe consequences of a complete absence of hepatic portal perfusion. In Abernethy malformation type Ib, the splanchnic circulation bypasses the liver entirely, impairing clearance of gut-derived toxins, bacteria, and inflammatory mediators [14]. In isolation, this can lead to metabolic or neurologic symptoms. However, when compounded by acquired thromboses, as in our patient, the result is a dramatic increase in venous pressure, intestinal wall congestion, and eventual ischemia. The development of bowel perforation, peritonitis, and systemic inflammatory response syndrome can be understood as a downstream effect of both mechanical and immunological compromise. The histological finding of IgG4-positive plasma cells in mesenteric lymph nodes, in the absence of elevated serum IgG4 or systemic disease features, may reflect a localized immune activation in response to chronic venous congestion and antigenic exposure [15]. This represents a novel observation in the context of CPSS.

Compared to the five published cases, our report is the only one to document a combination of trisomy 21, Abernethy malformation type Ib, splanchnic thromboses, bowel perforation, open abdomen management, and IgG4-positive lymphadenopathy. It is also the only case in which all standard interventional strategies were contraindicated, necessitating a purely supportive and surgical approach. Despite this, the patient achieved clinical stabilization, although vascular abnormalities persisted on follow-up.

## Conclusions

This case illustrates a rare and complex presentation of Abernethy malformation type Ib in a young adult with trisomy 21, uniquely complicated by extensive splanchnic vein thromboses, bowel ischemia with perforation, polymicrobial sepsis, and localized IgG4-positive lymphadenopathy. In contrast to previously reported cases, our patient exhibited a multifactorial pathophysiological cascade that precluded standard interventional treatments. The complete absence of portal venous inflow, combined with acquired thromboses, resulted in severe venous congestion and gastrointestinal compromise. Although the patient stabilized clinically under supportive and surgical management, persistent vascular abnormalities remain, and the risk of future complications, including progressive hepatic dysfunction, hepatic encephalopathy, or recurrent infection, must be considered. In this context, liver transplantation may represent the only curative option in selected patients with Abernethy malformation type I and non-correctable anatomy. This case expands the known clinical spectrum of CPSS and underscores the importance of early recognition, accurate anatomical characterization, and individualized, long-term planning in syndromic patients with this condition.
